# Acrolein exposure from electronic cigarettes

**DOI:** 10.1093/eurheartj/ehaa002

**Published:** 2020-03-25

**Authors:** Bernd Mayer

**Affiliations:** Department of Pharmacology and Toxicology, Institute of Pharmaceutical Sciences, University of Graz, Humboldtstraße 46/I, A-8010 Graz, Austria

**This commentary refers to ‘Short-term e-cigarette vapour exposure causes vascular oxidative stress and dysfunction: evidence for a close connection to brain damage and a key role of the phagocytic NADPH oxidase (NOX-2)’, by M. Kuntic *et al*., doi:10.1093/eurheartj/ehz772.**

In a recent study, Kuntic *et al*.^1^ reported that inhaled acrolein may be the main culprit for their observations of acute adverse effects of e-cigarette use on vascular function, caused by oxidative stress in blood vessels. According to Table S3 of their manuscript, the aerosol generated for the current study contained 0.0032 µg of acrolein per puff. Assuming an average of 600 puffs per day this corresponds to a daily intake of 0.96 µg of acrolein. For a human with 60 kg bodyweight, this amount is 450-fold lower than the daily dose accepted by the WHO (7.5 µg/kg) and below the estimated daily intake from other sources.^2^^,^^3^ The WHO threshold refers to oral intake of acrolein, which may result in reduced bioavailability due to liver metabolism but is unlikely to compensate for a 450-fold difference. Moreover, a study measuring biomarkers of toxin exposure found that e-cigarette users were having similar exposure to acrolein [measured as *N*-acetyl-*S*-(3-hydroxypropyl)-l-cysteine, an established biomarker of acrolein exposure] as former smokers using nicotine replacement therapies.^4^ Thus, exposure to acrolein from e-cigarette use appears to be too low to cause any adverse health effects.

Kuntic *et al*. report on vascular oxidative stress in response to acrolein, shown as increased dihydroethidium (DHE) staining (a marker for superoxide formation) of mouse aortic cryosections that had been incubated with 1–10 µM acrolein (Figure 5E in their manuscript). Assuming a plasma volume of 3 L, 100% bioavailability, and accumulation of acrolein in the bloodstream without elimination or metabolism for 24 h, daily intake of 0.96 µg of acrolein from e-cigarette use would result in a plasma concentration of 0.0057 µM. Acrolein had no effect on DHE staining at a concentration of 0.1 µM, which is more than 17-fold higher than the concentration mentioned above. The 1 µM concentration that did increase DHE staining is more than 170-fold higher than the upper limit of the plasma concentration calculated with the unrealistic assumption that the aldehyde accumulates in blood for 24 h without metabolism or elimination.^5^

In conclusion, the findings by Kuntic *et al*. about the effects of acrolein exposure on inducing vascular oxidative stress and dysfunction are of questionable clinical relevance for e-cigarette users.

**Conflict of interest:** none declared. 
